# Coupling wastewater treatment, biomass, lipids, and biodiesel production of some green microalgae

**DOI:** 10.1007/s11356-023-25628-y

**Published:** 2023-02-03

**Authors:** Mostafa M. El-Sheekh, Hamdy R. Galal, Amal SH. H. Mousa, Abla A. M. Farghl

**Affiliations:** 1 Botany Department, Faculty of Science, Tanta University, Tanta, 31527 Egypt; 2 Botany and Microbiology Department, Faculty of Science, South Valley University, Qena, Egypt

**Keywords:** Chlorophyta, Sewage wastewater, Bioremediation, Fatty acids composition, Green energy, Pollutants bioremoval

## Abstract

This study demonstrates the combination of wastewater treatment and green microalgae cultivation for the low-cost production of lipids as a feedstock for biodiesel production. Three green microalgal species were used: *Chlamydomonas reinhardtii*, *Monoraphidium braunii*, and *Scenedesmus obliquus.* Nutrient, heavy metals and minerals removal, biomass productivity, carbohydrate, protein, proline, lipid, and fatty acids methyl ester (FAMEs) contents besides biodiesel properties were evaluated. The results showed that all algal species were highly efficient and had the potential to reduce nitrate, ammonia, phosphate, sulfate, heavy metals (Zn^2+^, Cu^2+^, Mn^2+^, and Fe^2+^), calcium, magnesium, sodium, and potassium after 10 days of algal treatment compared to initial concentrations. The removal efficiency of these parameters ranged from 12 to 100%. The growth rates of *M*. *braunii* and* S*. *obliquu*s cultivated in wastewater were significantly decreased compared to the control (synthetic medium). In contrast,* C*. *reinhardtii* showed the highest growth rate when cultivated in sewage water. Wastewater could decrease the soluble carbohydrates and protein content in all tested algae and increase the proline content in *M*. *braunii* and *S*. *obliquus*. In wastewater culture, *M. braunii* had the highest lipid productivity of 5.26 mg L^−1^ day^−1^. The fatty acid profiles of two studied species (*C. reinhardtii* and *M. braunii*) revealed their suitability as a feedstock for biodiesel production due to their high content of saturated fatty acids, representing 80.91% and 68.62% of the total fatty acid content, respectively, when cultivated in wastewater. This study indicated that wastewater could be used to modify biomass productivity, lipid productivity, and the quantity of individual fatty acids in some algae that affect biodiesel quality to achieve international biodiesel standards.

## Introduction

Bioremediation is the process of treating wastewater with naturally existing bacteria and other elements of the environment. Additionally, it is cheaper than alternative technologies for disposing of hazardous waste (Vidali 2001). The environmentally friendly and economically practical approach of nutrient removal and biomass generation is offered by microalgae in wastewater treatment (Fawzy and Issa 2016).

Many studies showed that using wastewater can produce not only wastewater reuse but also a great amount of biomass transformation and production, particularly in the microalgae biofuels production and other uses with excellent application prospects (Zhou et al. 2018; Hernández-García et al. 2019). Microalgae engross many nutrients since they need a high amount of phosphorus and nitrogen to synthesize 45–60% of microalgae dry-weight proteins, nucleic acids, and phospholipids.

Sewage and wastewater must be treated since heavy metals are toxic to flora and fauna, and their occurrence in the environment is a significant problem (Adefila et al. 2010). The use of microalgae in heavy metal phycoremediation is a growing trend because of their numerous advantages, including their widespread availability, low costs, high metal removal efficiency, and eco-friendly nature (Leong and Chang 2020; Touliabah et al. 2022; Znad et al. 2022). According to Wang and Davidson (2010), algae may absorb organic contaminants entering cells, such as lipids and carbohydrates, resulting in an environmentally friendly pollution reduction.

In wastewater, some types of algae grow slowly. The wastewater’s chemical makeup or excessive heavy metal levels may cause an inhibiting effect (El-Sheekh et al. 2005, 2016b). The primary characteristic of the technology is the capacity of algae to grow in wastewater to efficiently assimilate organic carbons and inorganic nutrients and achieve maximal algal biomass, effective nutrient removal, and lipid accumulation, which can then be turned into biodiesel (Perez-Garcia et al. 2011). Microalgae grown in wastewater can accumulate lipids ranging from low (10% dry weight) to moderate (25–30% DW) and can provide up to 80% of DW (Abou-Shanab et al. 2013).

Algae have a yearly oil yield that is 7 to 31 times more than palm oil due to their ability to accumulate lipids and their high photosynthetic production (Hu et al. 2008; El-Sheekh et al. 2017a). A potential renewable energy source that could replace fossil fuel without compromising the availability of food for people is biodiesel produced from microalgae (Chisti 2007). One alternative fuel is biodiesel (monoalkyl esters), produced by transesterifying triglyceride oil with monohydric alcohols. Based on previous reports, biodiesel made from canola, soybean, palm, sunflower, and algae can be used as a diesel fuel substitute (Mostafa et al. 2012)*.*

The total biodiesel yield depends on the algal strain’s lipid content and growth rate. In producing biodiesel, the economic biomass production of microalgae must be considered; hence, microalgal species with a high lipid content and cell growth are utilized (Lv et al. 2010).

Factors influencing fuel qualities such as viscosity, specific gravity (SG), cetane number (CN), cloud point (CP), iodine value (IV), and greater heating value (HHV) may be related to fatty acid (FA) chain length and the unsaturation degree in fatty acid methyl ester (FAME) (Knothe 2011; Hoekman et al. 2012). As the chain length increases, CN, Vis, and CP increase, and as unsaturation increases, they decrease (Francisco et al. 2010; Tan and Lin 2011; Hoekman et al. 2012).

On the contrary, SG, IV, and HHV decrease with the increase of chain length and increase with the increase of unsaturation (Refaat 2009). Long chain length and low unsaturation are preferred to achieve low-temperature performance and oxidative stability in biodiesel fuel (Refaat 2009; Hoekman et al. 2012).

The current study demonstrates the bioremoval capacity of three green microalgae: *Chlamydomonas reinhardtii*, *Monoraphidium braunii*, and *Scenedesmus obliquus* from sewage wastewater with simultaneous lipid and fatty acid (FA) accumulation to provide low-cost and environmentally friendly biodiesel production technology.

## Materials and methods

### Algal isolation and culturing

The unicellular green microalgae, *Chlamydomonas reinhardtii*, *Monoraphidium braunii,* and *Scenedesmus obliquus*, were isolated from a water sample collected from South Valley University. Liquid and solid Beijerinck’s nutritive media were used for the cultivation and isolation of *C. reinhardtii* and *M*. *braunii* (Stein 1966), and blue-green 11 medium was used for *Scenedesmus obliquus* according to Song et al. (2013). The growth of algal species was evaluated and identified using a compound microscope (Leica DM500) (Prescott-Allen and Prescott-Allen 1982).

### Algal growth and culture conditions

In separate 500-mL culture flasks with a nutritive media, *Chlamydomonas reinhardtii*, *Monoraphidium braunii*, and *Scenedesmus obliquus* axenic algal samples were cultured*.* The cultures were exposed to 100 µmol photons m^−2^ s^−1^ of cool-white fluorescent light while being incubated at 25 °C ± 1 °C. The dry air was delivered to the algal cultures (Lorenzen 1964) to maintain algae suspension without mechanical stress, provide CO_2_ needed for photosynthesis, and prevent cells from settling at the bottom of the containers (Persoone et al. 1980). The algae were routinely sub-cultured and preserved in ideal circumstances.

### Green microalgae treatment of sewage wastewater sample

#### Water sampling and experimental design

A sample of sewage water was taken from the sewage station in Qena, Egypt, called Al-Salhya. The algal cells were inoculated in 10-L glass tanks containing a nutritive medium. Sewage water samples were inoculated with algae at a concentration of 5% by adding algal cell cultures. Sewage wastewater was treated with algae for 10 days under illumination and aeriation. The algal mats were precipitated by centrifuging the treated sewage water. The properties of the sewage water were determined using a supernatant. The precipitate algal mats were used to investigate the effect of sewage water on the tested algae.

#### Chemical analysis of sewage wastewater

A preliminary analysis of sewage water was performed before the inoculation of algae. The total content was filtered to eliminate algae at the final step in each flask and then used to measure various parameters, including nitrate, ammonia, sulfate, and phosphate based on standard methods (American Public Health Association (APHA) 2005). Heavy metals (Fe^2+^, Cu^2+^, Zn^2+^, and Mn^2+^), calcium, magnesium, sodium, potassium, biological oxygen demand (BOD), and chemical oxygen demand (COD) were evaluated by atomic absorption (spectrometer: MESLO). The following equation was used to determine the percentage of metal ion biosorption.

$$\mathrm{Biosorption\;\%}=[({\mathrm{C}}_{\mathrm{i}}-{\mathrm{C}}_{\mathrm{f}})/{\mathrm{C}}_{\mathrm{i}}\times 100$$

where *C*_*i*_ is the initial concentration and *C*_*f*_ is the final concentration.

#### Effect of sewage wastewater on the growth and some metabolites of green microalgae

The effect of sewage water on the growth and metabolites of the tested algae was investigated using precipitate algal mats (algal cells).

#### Biomass assay

The optical density of the culture for microalgal growth was measured at 560 nm (OD560) using a spectrophotometer and by determining the algal cellular dry weight (CDW). Biomass productivity was determined according to Abomohra et al. (2013).

$$\mathrm{Biomass\;productivity}\left(\mathrm{g }{\mathrm{L}}^{-1}{\mathrm{day}}^{-1}\right)=({\mathrm{CDW}}_{\mathrm{L}}-{\mathrm{CDW}}_{\mathrm{E}})/\left({\mathrm{t}}_{\mathrm{L}}-{\mathrm{t}}_{\mathrm{E}}\right),$$

where *CDW*_*E*_ indicates the *CDW* (g L^−1^) at days of the early exponential phase (*t*_*E*_) and *CDW*_*L*_ at days of the late exponential phase (*t*_*L*_).

### Biochemical assessment of cell constituents

Using a spectrophotometer, pigment fractions (Chl. a, Chl. b, and carotenoids) were estimated in accordance with the method of Metzner et al. (1965).

Pigment fractions were measured by µg/mL algal suspension. Soluble saccharides were calorimetrically determined using the anthrone sulfuric acid method, according to Badour (1959). The soluble proteins were calculated using a calibration curve made using serum albumin and quantified using a spectrophotometer in accordance with the procedure outlined by Lowry et al. (1951). According to the Bates et al. (1973) method, proline was measured spectrophotometrically at 520 nm. Absorbance was calculated on a dry weight basis as mg proline/gm DW using a standard curve.

### Determination of lipid content

The modified protocol of El-Sheekh et al. (2017b) was used to extract total lipids from algae. In brief, cells were homogenized with chloroform/methanol (2/1). After dispersion, the mixture was stirred for 20 min at room temperature in an orbital shaker.

A 0.2 volume of 0.9% NaCl solution was used to wash the solvent. Centrifugation was used to divide the homogenate into two phases. Under an argon stream, the lipid extracts were dried in pre-weighted glass vials for 30 min at 80 °C, cooled in a desiccator, and then weighed.

Lipid productivity calculation.

Lipid productivity was determined based on Andrade and Costa (2007) and modified by Abomohra et al. (2013).

$$\mathrm{Lipid\;productivity}\left(\mathrm{mg }{\mathrm{L}}^{-1}{\mathrm{d}}^{-1}\right)=({\mathrm{L}}_{\mathrm{D}}-{\mathrm{L}}_{0})/({\mathrm{T}}_{\mathrm{D}}-{\mathrm{T}}_{0})$$

where *L*_0_ and *L*_*D*_ represent the total lipid (mg L^−1^) of cultivation on the first day (*T*_0_) and the desired days phase (*T*_*D*_), respectively.

### Fatty acids profile

The lipids and fatty acids methyl esters (FAMEs) transmethylation were extracted as described by Radwan (1978) using the GC–MS system (Agilent Technologies) equipped with a gas chromatograph (7890B) and mass spectrometer detector (5977A) at Central Laboratories Network, National Research Centre, Cairo, Egypt. The GC was equipped with a DB-WAX column (30 m × 250 μm internal diameter and 0.25 μm film thickness). By contrasting the spectrum fragmentation pattern with those found in the Wiley and NIST Mass Spectral Library data, the various constituents were distinguished.

### Assessment of biodiesel properties

The produced biodiesel quality was estimated from the fatty acid profile by chemical and physical properties calculations, including the average degree of unsaturation (ADU, %), cetane number (CN), iodine value (IV, g I2 100 g^−1^ oil), kinematic viscosity (KV, mm^2^ s^−1^), specific gravity (SG, kg^−1^), cloud point (CP, °C) higher heating value (HHV, MJ kg^−1^), long chain saturation factor (LCSF, %wt.), and cold filter plugging point (CFPP, C) from Eqs. (1)–(9) (Hoekman et al. 2012; Ma et al. 2013).

1 $$\mathrm{ADU}=\sum N\times Mf$$

2 $$\mathrm{CN}=6.6684 \mathrm{ADU}+62.876$$

3 $$\mathrm{IV}=74.373 \mathrm{ADU}+12.71$$

4 $$\mathrm{KV}=-0.6313 \mathrm{ADU}+5.2065$$

5 $$\mathrm{SG}=0.0055 \mathrm{ADU}+0.8726$$

6 $$\mathrm{CP}=-3.356 \mathrm{ADU}+19.994$$

7 $$\mathrm{HHV}=1.7601 \mathrm{ADU}+38.534$$

8 $$\mathrm{LCSF}=\left(0.1\times \mathrm{C}16:0\right)+\left(0.5\times \mathrm{C}18:0\right)+\left(1.0\times \mathrm{C}20:0\right)+\left(1.5\times \mathrm{C}22:0\right)+(2.0\times \mathrm{C}24:0)$$

9 $$\mathrm{CFPP}=(3.1417\times (\mathrm{LCSF})-16.477$$

where the number of carbon–carbon double bonds in the corresponding fatty acid is *N*, the mass fraction of each fatty acid is *Mf*, and the weight percentage of each corresponding fatty acid are C16:0, C18:0, C20:0, C22:0, and C24:0.

### Statistical analysis

The data were statistically examined using the SPSS statistical computer program and tested using a one-way analysis of variance (version 23).

## Results and discussion

### Reduction of nutrients from wastewater by green microalgae

The obtained data demonstrated that after algal treatment of the sewage wastewater, nutrient concentrations such as nitrate (NO_3_^−^), ammonia (NH_4_^+^), phosphate (PO_4_^3−^), and sulfate (SO_4_^2−^) significantly reduced (Table 1).

**Table 1:** The removal efficiency of some nutrients (mg/L), heavy metals (µg/L), minerals, BOD, and COD (mg/L) from wastewater using *C*. *reinhardtii*, *S*. *obliquus*, and *M*. *braunii*

Parameters	Initial concentrations	After treatment					
		*M*. *braunii*	% of removal	*C. reinhardtii*	% of removal	*S*. *obliquus*	% of removal
NO_3_^−^ (mg/L)	72.54 ± 0.1	48.06^*^ ± 0.1	34.0	31.37^*^ ± 0.2	57.0	47.47^*^ ± 0.2	35.0
NH_4_^+^ (mg/L)	20.23 ± 0.2	9.44^*^ ± 0.01	53.0	6.22^*^ ± 0.1	69.0	5.86^*^ ± 0.2	71.0
PO_4_^3−^ (mg/L)	18.01 ± 0.01	4.86^*^ ± 0.07	73.0	16.73^*^ ± 0.01	7.0	6.79^*^ ± 0.2	62.0
SO_4_^2−^ (mg/L)	60 ± 0.01	50.0^*^ ± 0.1	17.0	47^*^ ± 0.02	22.0	53^*^ ± 0.05	12.0
Fe^2+^ (µg/L)	108 ± 0.5	0.0	100.0	13.7* ± 0.3	87.0	0.0	100.0
Cu^2+^ (µg /L)	78 ± 0.2	0.0	100.0	25* ± 0.5	68.0	0.0	100.0
Zn^2+^ (µg /L)	43 ± .01	0.0	100.0	17* ± 0.2	60.0	19.1* ± 0.7	56.0
Mn^2+^ (µg /L)	37 ± 0.6	5.0* ± 0.16	86.0	0.0	100.0	0.0	100.0
Ca^2+^ (mg/L)	98.23 ± 0.2	53.0^*^ ± 0.00	46.0	39^*^ ± 0.06	60.0	36^*^ ± 0.6	63.0
Mg^2+^(mg/L)	116 ± 0.1	80.0^*^ ± 0.06	31.0	69^*^ ± 0.02	41.0	89^*^ ± 0.06	23.0
Na^+^(mg/L)	47.4 ± 0.04	10.18^*^ ± 0.1	79.0	34^*^ ± 0.00	28.0	18.05^*^ ± 0.02	62.0
K^+^(mg/L)	18.3 ± 0.02	3.75^*^ ± 0.1	80.0	10.35^*^ ± 0.2	43.0	9.3^*^ ± 0.02	49.0
BOD (mg/L)	150 ± 0.05	28^*^ ± 0.6	81.33	15^**^ ± 0.04	90	21^*^ ± 0.02	86
COD (mg/L)	240 ± 0.03	67^*^ ± .01	72.1	28.8^*^0.06	88	74.4^*^ ± 0.14	69

The reduction efficiency of nitrate**,** ammonia**,** phosphate, and sulfate reached 57%, 69%, 7%, and 22% in the cultures of *C*. *reinhardtii* grown in the wastewater. A reduction of 35%, 71%, 62%, and 12% was detected by using *S*. *obliquus*. The removal efficiency by *M. braunii* accounted for 34%, 53%, 73%, and 17%, compared to the control (initial concentrations).

A similar observation was recorded by Kshirsagar (2014) and El-Sheekh et al. (2016a), who showed that the amount of nutrients consumed during algal growth or absorbed into tissues of algae affected the removal efficiency of nutrients in wastewater. Nitrogen is a significant macro element in microalgae, accounting for 1 to 10% of total biomass, and it is also an important factor in lipid content regulation within algal cells (Chisti 2008). The common bioavailable nitrogen compounds assimilated by microalgae are ammonium (NH_4_^+^) and nitrate (NO_3_^−^). Ammonium is preferred as a source of nitrogen for the microalgae, and when this is available, no other nitrogen source will be used (Abdel-Raouf et al. 2012). The use of phosphorus for algal growth during phycoremediation results in phosphorus removal. Phosphorus, which is used for the formation of phospholipids, adenosine triphosphates, and nucleic acids in algal cells, is absorbed as inorganic orthophosphate, and this active process requires energy (Emparan et al. 2019). Microalgae can absorb excess phosphorus, which are then granulated into polyphosphate (volutin) and stored within the cells. In the absence of available phosphorus, these reserves can be sufficient for a prolonged growth (Woertz et al. 2009).

### Removal of heavy metals from sewage wastewater by green microalgae

The data in Table 1 exhibited that heavy metal concentrations were significantly reduced after biotreatment. From the data, *M. braunii* culture could remove 100% (Fe^2+^, Cu^2+^, and Zn^2+^) and 86% (Mn^2+^) from wastewater. The reduction accounting for 56% (Zn^2+^) and 100% (Fe^2+^, Cu^2+^, and Mn^2+^) were observed using *S*. *obliquus* as biosorbent. The removal efficiency of Fe^2+^, Cu^2+^, Zn^2+^, and Mn^2+^ reached 87%, 68%, 60%, and 100%, respectively, in C. *reinhardtii* cultures as compared to the control (initial concentration).

In this study, green microalgae were highly effective in removing heavy metals from sewage water. The results demonstrate that *M*. *braunii* and *S*. *obliquus* were more capable of bioremoval of heavy metals than *C*. *reinhardtii*. In addition, the types of heavy metals and algae utilized affected the removal effectiveness of heavy metals. The surface of cell adsorption and cytoplasmic attaching to compounds such as phytochelatins, metallothioneins, as well as intracellular ligands may be responsible for the metal ion accumulation (Peña-Castro et al. 2004). The large surface area and high binding affinity of algae contribute to their efficiency in removing heavy metals from wastewater (Sattayawat et al. 2021).

Algae cell walls contain a variety of functional groups, including sulphydryl, carboxyl, amino, phosphoryl, and hydroxyl, which give the cell surface a negative charge. Cationic metal ions make up the majority of water ions; thus, they are adsorbed onto the surface of cells (Romera et al. 2006). The different microalgal species used in this study indicated the ability to remove heavy metals from a sewage water sample. This observation has been emphasized by RATH (2012), who stated that algal cells accumulated higher metal concentrations when grown in media with high metal levels.

### Reduction of some minerals from sewage wastewater by green microalgae

Table 1 showed that mineral content decreased markedly in the sewage sample because of algal treatments. The reduction efficiency of calcium, magnesium, sodium, and potassium reached 60%, 41%, 28%, and 43%, respectively, compared to the control in *C*. *reinhardtii* cultures grown in the wastewater. In the case of* S*. *obliquus* treatment, the removal efficiency of Ca^2+^, Mg^2+^, Na^+^, and K^+^ from wastewater was 63%, 23%, 62%, and 49%, respectively, compared to the control (initial concentration).

The reduction of Ca^2+^ (46%), Mg^2+^ (31%), Na^+^ (79%), and K^+^ (80%) was observed by using *M*. *braunii* as a biosorbent. This finding is consistent with that obtained by Azab (2002) and El-Sheekh et al. (2016a), who stated that the algal application for wastewater treatment resulted in varying percentages of mineral reduction. In this regard, Rao et al. (2011) found that the utilization of *C. vulgaris* during phycoremediation significantly reduced magnesium levels and moderately decreased potassium levels. However, they observed a decrease in sodium levels and a significant decrease in calcium.

### Removal of biological oxygen demand and chemical oxygen demand

Treated wastewater resulted in a significant reduction in BOD and COD levels. Substances that can be degraded biologically and consume dissolved oxygen in the treatment during the cultivation period were expressed as the BOD indicator. The BOD level was reduced to 90%, 86%, and 81.33% by *C*. *reinhardtii*, *S*. *obliquus*, and *M*. *braunii*, respectively (Table 1). El-Sheekh et al. (2016a) claim that the value of BOD represents the degree of toxicity of wastewater and that utilizing *Chlorella* species resulted in a 90% reduction in BOD of sewage water. BOD in wastewater represents the respiratory requirements of the algae and bacteria that absorb the organic matter, and an excess of BOD typically reduces the dissolved oxygen (Abdel-Raouf et al. 2012). Zhang et al. (2008), Azarpira et al. (2014), and El-Sheekh et al. (2016a) stated very high BOD reductions using various algal species, proving that microalgae are the best choice for improving physicochemical parameters and wastewater purification. The COD level was also reduced to 88%, 72%, and 69% by *C*. *reinhardtii*, *S*. *obliquus*, and *M*. *braunii*, respectively (Table 1). Similar observations were recorded by Azarpira et al. (2014) and El-Sheekh et al. (2016a). The chemical oxidation of carbon in organic pollutants, which releases carbon dioxide, lowers COD levels. Additionally, faster biodegradation and bioconversion of organic matter because algae may also have a role. In the present study, the BOD and COD levels were higher, reduced to 90% and 88% by *C*. *reinhardtii*, showing COD and BOD’s best removal capacity from wastewater.

### Influence of wastewater on the growth rate of green microalgae

**Fig. 1:**
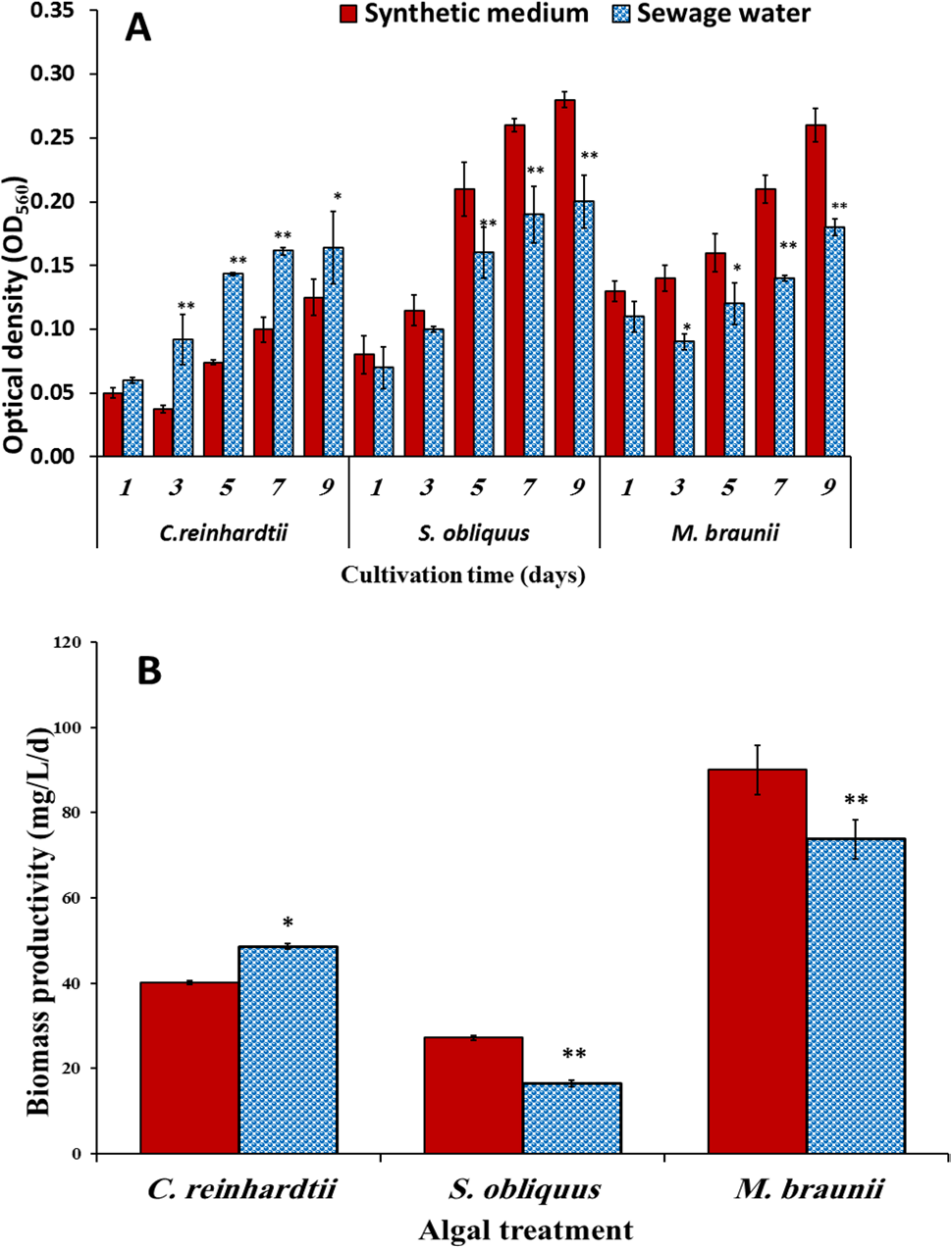
Variation of optical density (**A**) and biomass productivity BP (**B**) of some green microalgae cultivated in synthetic medium (control) and sewage wastewater. Error bars show the SD for three measurements

The effect of wastewater on the growth rate of green microalgae was investigated using the density of algal cells (measured as optical density, OD_560_) and cellular dry weight (CDW) (biomass productivity) after 10 days of incubation. The optical density of* M*. *braunii* and* S*. *obliquu*s was significantly reduced in wastewater; it was reduced by 31% and 29% when compared to the control (synthetic medium), respectively (Fig. 1). As opposed to that, the optical density of *C*. *reinhardtii* considerably increased in wastewater compared to the control; it was increased by 23% of control The productivity of biomass of *M*. *braunii* and S. *obliquus* in the late exponential phase decreased from 90.0 and 27.2 mg L^−1^ d^−1^ in synthetic medium to 73.75 and 16.45 mg L^−1^d^−1^ in wastewater, respectively (Fig. 1). In contrast, *C*. *reinhardtii* demonstrated the highest biomass productivity in wastewater (48.62 mg L^−1^ day^−1^) corresponding to (40.2 mg L^−1^ day^−1^) in synthetic medium (control).

This finding is consistent with El-Sheekh et al. (2005), who found that microalgae grew more rapidly in sewage wastewater than in Allen’s medium. Available organic matter, phosphorus, and nitrogenous compounds may cause microbial growth. According to Park et al. (2018), the growth of microalgae and biomass are closely related to the photosynthesis occurrence and the subsequent production of energy; thus, they may serve as a biomarker of stress and an important way to express the ecological success of algal strains in adapting to its natural inhabitants. This result also agrees with that of Galgale et al. (2014) connected to the inhibition of wastewater effect to its high salinity levels, which may result in plasmolysis and loss of algal cell activity. Furthermore, a factor that prevented algae growth was a high ammonia concentration in sewage water. The growth of studied algae may be limited due to significant levels of heavy metals in wastewater. Heavy metals were identified as a source of toxicity in wastewater in this context (Tiruneh et al. 2014).

### Influence of sewage wastewater on the pigments of green microalgae

**Fig. 2:**
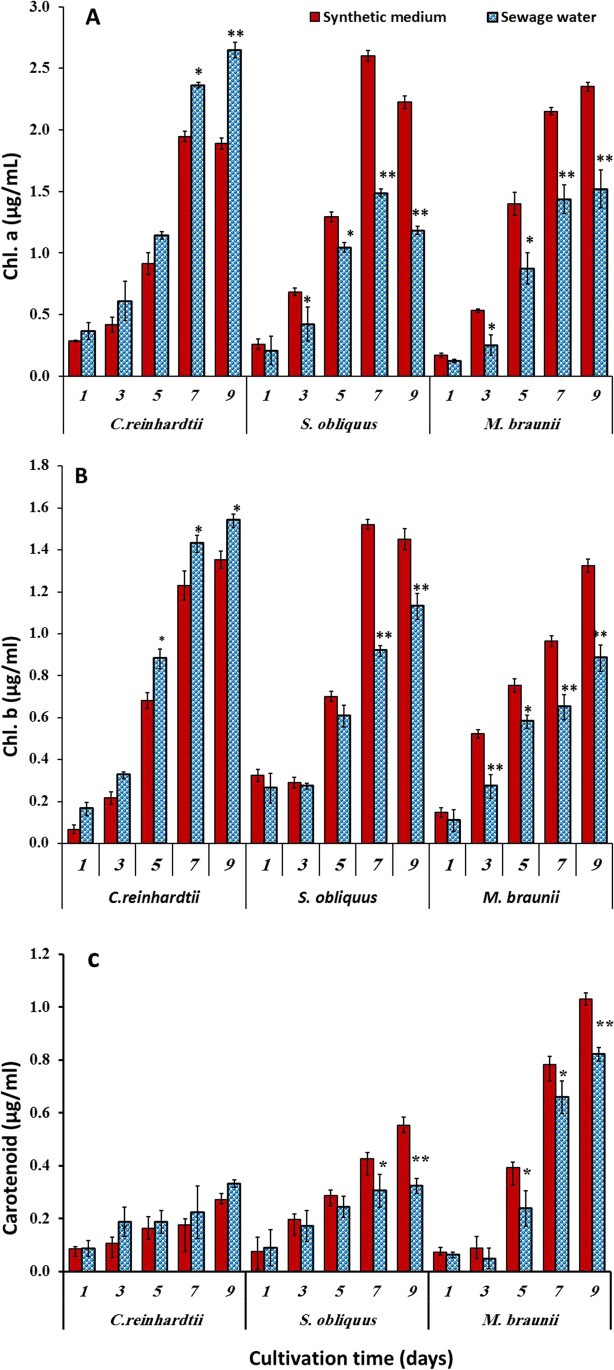
Variation of Chl. a (**A**), Chl. b (**B**), and carotenoid (**C**) contents of some green microalgae cultivated in synthetic medium (control) and sewage wastewater. Error bars show the SD for three measurements

Figure 2 revealed that the wastewater was responsible for the increased Chl. a, Chl. b, and carotenoids in *C*. *reinhardtii* reach 40%, 14%, and 23%, respectively, compared to the control (standard medium). In the case of *S. obliquus* and *M*. *braunii*, cultures observed that Chl. a, Chl. b, and carotenoid values are decreased markedly when cultivated in wastewater. The greatest inhibition effect of Chl. a and carotenoid contents of *S. obliquus* were 47% and 41% of the control (synthetic media), respectively (Fig. 2).

The reduction in pigment contents in *S. obliquus* and *M. braunii* revealed that these algae used the standard medium as a source of nutrition rather than sewage water because sewage water’s chemical composition differs from synthetic media. The quantity of internal metals and the amount of metals bonded to the surface of the algal cells were connected to the growth inhibition (Ma et al. 2003). This result might be attributed to the effect of wastewater on the photosynthetic system, which degrades the pigment content of cells. This observation was consistent with the findings of El-Sheekh et al. (2016b) and El-Otify et al. (2011), who linked the influence of heavy metals to the chlorophyll damage on the thylakoid membranes.

### Influence of sewage wastewater on soluble carbohydrate, soluble protein, and proline contents of green microalgae

Treatments with sewage water exerted a significant decrease in the content of soluble carbohydrates and proteins of *C*. *reinhardtii*, *S. obliquus*, and *M*. *braunii*, reaching 69.9%, 4.12%, 45.08%, 24.23%, 35.65, and 51.8% as compared to the control cultures grown in synthetic media (Table 2). The results shown in Table 2 cleared that proline content in *M*. *braunii* and *S*. *obliquus* grown in sewage water significantly increased by 92% and 153% more than that in synthetic medium. In contrast, sewage water decreased proline content in *C. reinhardtii* by 21.23% compared to synthetic medium (control). In general, the carbohydrate content of *C*. *reinhardtii* cells was more affected by sewage water treatment than other microalgae.

**Table 2:** Soluble carbohydrates, proteins and proline content of *C*. *reinhardtii*, *S*. *obliquus*, and *M*. *braunii* cultivated in wastewater and synthetic medium (control) after 10 days of incubation

Algal species	Treatment	Carbohydrates	%	Proteins	%	Proline	%
*C***. ***reinhardtii*	Control	63.7 ± 1.5	100	69.4 ± 2.0	100	33.1 ± 1.1	100
	Sewage water	19.14^**^ ± 0.15	69.95	52.58^**^ ± 0.9	24.23	26.07^**^ ± 0.4	21.24
*S***. ***obliquus*	Control	19.4 ± 5.2	100	11.5 ± 2.03	100	2.8 ± 0.33	100
	Sewage water	18.6 ± 5.9	4.12	7.4^*^ ± 2.7	35.65	7.1^**^ ± 13.12	153
*M***. ***braunii*	Control	22.14 ± 2.9	100	23.28 ± 0.55	100	1.23 ± 0.17	100
	Sewage water	12.16^**^ ± 1.8	45.08	11.22^*^ ± 9.5	51.8	2.37 ± 1.3	92.68

Similar to this, El-Sheekh et al. (2016b) found that wastewater caused green microalgae cultures to contain less carbohydrates than control cultures grown in synthetic media. This decrease is a result of heavy metals’ inhibition of photosynthetic activity and increased nitrogen metabolism brought on by the use of carbohydrates as substrates. Kathryn et al. (2013) reported that the difference in the chemical composition between the well-balanced medium and wastewater caused reductions in carbohydrate and protein contents. According to Tripathi and Gaur (2006), the toxicity of these metals on enzymes responsible for protein synthesis causes the accumulation of protein inhibited by metal concentrations. Soluble protein is one of the indicators of both reversible and irreversible changes in an organism’s metabolism, which is known to react to a number of stressors, including natural and xenobiotics. Proline helps to cleanse sewage water, acts as an antioxidant, and scavenges free radicals in the presence of metal stress (Sharma and Dietz 2009). Proline’s enhanced production in plants indicates that it is responding to antioxidants by acting as a protein stabilizer, an O_2_ scavenger, and an inhibitor of -OH^−^ (Sharma and Dietz 2009). According to Fatma et al. (2007), higher metabolism and reduced oxidative loss lead to increased proline content as a result of metal stress-oriented toxicity.

### Influence of sewage wastewater on the total lipid content and lipid productivity of green microalgae

**Fig. 3:**
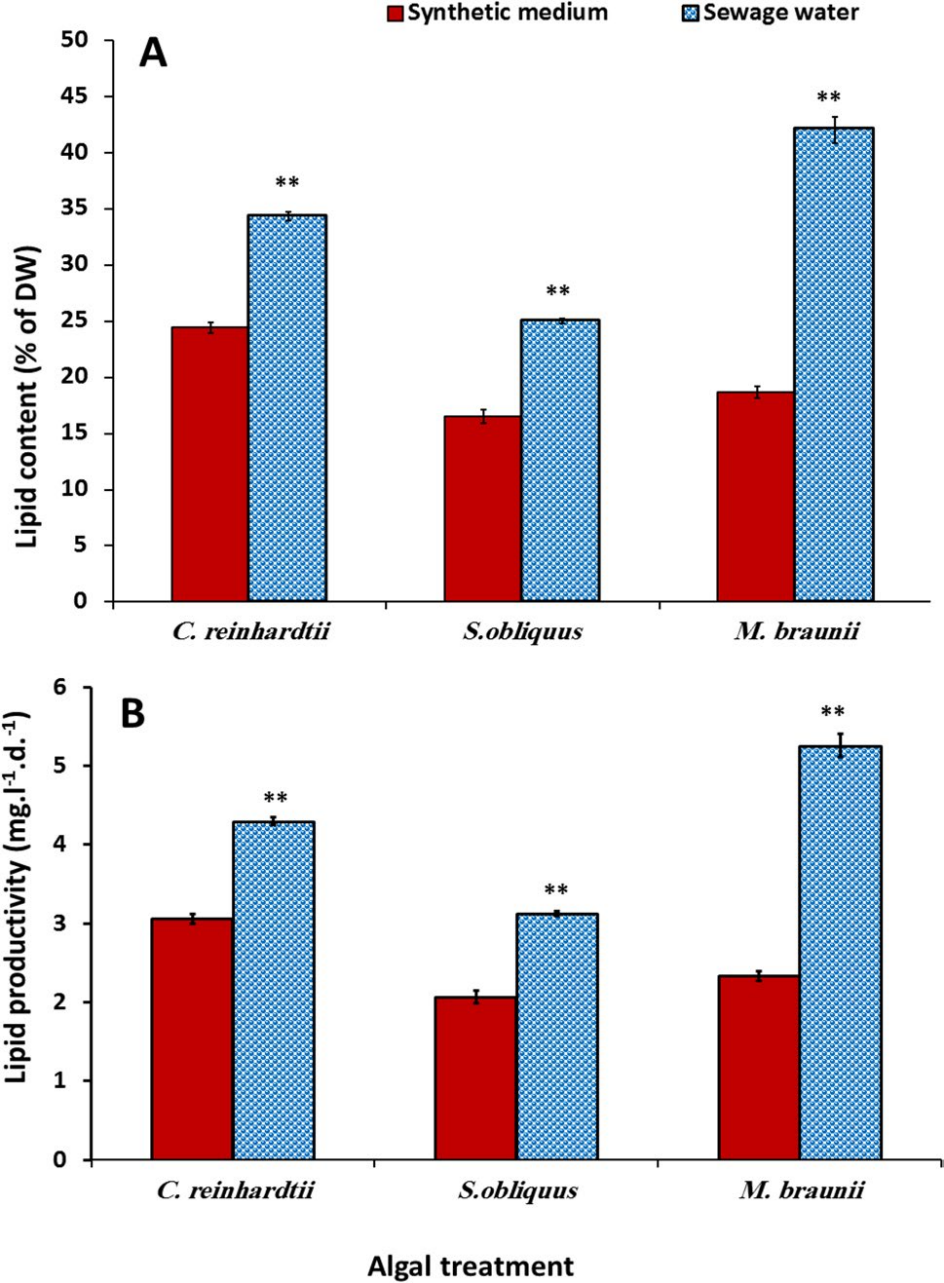
Variation of lipid content (**A**) and lipid productivity (**B**) of some green microalgae cultivated in synthetic medium (control) and sewage wastewater. Error bars show the SD for three measurements

The data in Fig. 3 showed that when these green microalgae were cultured in sewage wastewater, the total lipid content and lipid productivity of the three microalgae significantly increased compared to the synthetic medium (control). *M. braunii* had the highest lipid content and lipid productivity increase, which was more than twofold higher than the control (synthetic medium). This result could be due to differences in biotic stress responses among algal species and a severe lack of all required elements in secondary treated sewage water, particularly nitrogen, which increased the accumulation of lipids (Rodríguez-Palacio et al. 2022). The findings of this study were consistent with those of Widjaja (2009) and Mostafa et al. (2012), who revealed that the green microalgae (*C. vulgaris*) accumulated a high lipid content when grown in nitrogen-depleted conditions (0.02 mg/L nitrate). Furthermore, the current findings were in accordance with those of Shalaby et al. (2010) that the overproduction of carbon skeleton altered algal metabolism under salt stress conditions (with normal nitrate concentrations in culture media), which was partly directed toward the production of substances with beneficial roles in algal tolerance or defense mechanisms such as polyols, methylated amino acids, carbohydrate, and protein. One major criterion for selecting microalgae strains as a renewable source for biofuel production is high lipid content (El-Sheekh et al. 2017a). Xin et al. (2010) found that *Scenedesmus* sp. has high lipid levels under stress conditions, but that the lipid productivity and the microalgal biomass productivity were reduced due to the low growth rate.

According to Francisco et al. (2010), lipid content and biomass productivity are inversely related. In the current study, using the results of Francisco et al. (2010), at the late exponential phase, the lipid content and biomass output in the tested species *C. reinhardtii* were inversely correlated. The correlation study did not include *S*. *obliquus* and *M*. *braunii* because they showed relatively low biomass production and high lipid content in the wastewater.

### Fatty acids profile

The fatty acid composition of the three green microalgae cultivated in wastewater and the synthetic medium is displayed in Table 3. The results indicated that the two studied species (*C. reinhardtii* and *M. braunii*) have a high percentage of saturated fatty acids (80.91% and 68.62% of FAs, respectively) when grown in wastewater compared to synthetic medium, whereas there was a reduction in saturated fatty acid in *S. obliquus* (63.47% of TFAs) in wastewater corresponding to 72.24% of TFAs in the synthetic medium mainly composed of palmitic acid (C16:0), stearic acid (C18:0), and myristic acid (C14:0) which are the most abundant fatty acid. On the other hand, the largest concentration of monosaturated fatty acids (MUFAS), 17.71% of TFAs, was recorded in *M. braunii* grown in a synthetic medium, corresponding to 12.52% TFAs in wastewater due to the existence of palmitoleic acid (C16:1) and elaidic acid (C18:1). Finally, the total polyunsaturated fatty acids (PUFAS) in *C*. *reinhardtii*, *M*. *braunii*, and *S*. *obliquus* grown in wastewater increased to 12.0%, 13.27%, and 16.75% of TFAs, respectively, as compared to those in synthetic media (control) due to the presence of benzene propanoic acid (C17:3), linoleic acid (C18:2), and linolenic acid (C18:3).

**Table 3:** Fatty acid profiles of *C*. *reinhardtii*, *S*. *obliquus*, and *M*. *braunii* cultivated in wastewater and synthetic medium (control)

	*C***. ***reinhardtii*		*S***. ***obliquus*		*M***. ***braunii*	
	Synthetic medium	Sewage water	Synthetic medium	Sewage water	Synthetic medium	Sewage water
SFAs						
Myristic acid, C14:0	1.89	1.42	1.25	1.08	1.06	1.22
Methyl 12-methyltetradecanoate, C15:0	ND	0.45	ND	ND	ND	ND
Palmitic acid, C16:0	54.98	41.62	45.74	42.95	46.69	47.48
Stearic acid, C18:0	17.14	36.78	25.25	19.44	17.5	19.92
Phytanic acid, C19:0	0.6	0.64	ND	N.D	ND	ND
Ʃ SFAs	74.61	80.91	72.24	63.47	65.25	68.62
MUFA						
Palmitoleic acid, C16:1	1.17	1.62	2.54	2.93	2.49	2.49
Elaidic acid, C18:1	12.56	3.73	7.18	8.63	15.22	10.03
Ʃ MUFA	13.73	5.35	9.72	11.56	17.71	12.52
PUFAs						
Benzene propanoic acid, C17:3	2.78	7.62	10	7.79	2.57	5.63
Linoleic acid, C18:2	7.81	4.38	4.77	8.96	8.59	7.64
Linolenic acid, C18:3	1.07	1.73	3.27	8.22	5.88	5.58
Ʃ PUFAs	11.66	12	14.77	16.75	11.16	13.27

Values are given as percentages (%) of total fatty acids

*SFAs*, saturated fatty acids; *MUFA*, mono-unsaturated fatty acid; *PUFAs*, polyunsaturated fatty acids

These findings indicated a range in saturated and unsaturated fatty acid composition and a variation in fatty acid chain length between C-14 and C-19. Our findings were consistent with previous research demonstrating the removal of nutrients (TN and TP) by algal biomass and algal lipid content after cultivation in various municipal wastewater effluents (Abou-Shanab et al. 2014). The characteristics of biodiesel would be impacted by the chain length and saturation of fatty acids. Because of their high oxidation stability and avoiding cold flow, products with a high percentage of unsaturated fatty acids are not preferred in terms of quality (Krzemińska and Oleszek 2016).

Therefore, the transesterification process and biodiesel quality are positively affected by the large proportion of saturated fatty acids (Hu et al. 2008). According to the results, all microalgal species contain a high proportion of saturated fatty acids with C16–C18 carbon chains, which are highly recommended for the production of biodiesel, and only a small amount of linoleic acid (C18:3) is below 12%; their biomass is also suitable for the production of biodiesel from these microalgae species (Rodríguez-Palacio et al. 2022). Additionally, the high C16–C18 percentage enhances the performance and quality of biodiesel (Elshobary et al. 2020; Huo et al. 2020). Therefore, the fatty acid profile (FAMEs) produced by all species is suitable for synthesizing biodiesel based on the current research.

### Biodiesel quality

For sustainable biodiesel production, selecting microalgal species requires high lipid productivity and acceptable characteristics of the produced fatty acid methyl esters (FAMEs). Table 4 shows the main physicochemical properties of biodiesel of the green microalgae cultivated in wastewater and synthetic medium compared to the international standards (ASTM D6751 in the US and EN 14,214 in Europe). The KV, CN, IV, SV, and ρ values are estimated in *C*. *reinhardtii*, *S*. *obliquus*, and *M*. *braunii* stayed within the bounds of the international standards, low KV (1.13 mm^2^ s^−1^), the relatively high CN (60.15), low IV value (43.12 g I2 100 g^−1^ oil) and high SV (199.83 mg KOH g^**−1**^) detected in *C*. *reinhardtii*. The KV, CN, IV, and SV in *S*. *obliquus* and *M*. *braunii* with a small difference between those in *C*. *reinhardtii* and all estimated results were in line with the international standards recommendations.

**Table 4:** Estimated physicochemical properties of biodiesel for the biomass of *C*. *reinhardtii*, *S*. *obliquus*, and *M*. *braunii* cultivated in wastewater and the synthetic medium (control)

Parameters	*C*. *reinhardtii*		*S*. *obliquus*		*M*. *braunii*		*ASTM D6751*	*EN 14,214*
	Synthetic medium	Sewage water	Synthetic medium	Sewage water	Synthetic medium	Sewage water		
ADU	0.41	0.42	0.59	0.77	0.60	0.61	-	-
ʋ I (mm^2^s^−1^)	1.13	1.14	1.24	1.36	1.25	1.26	1.9–6.0	3.5–5.0
CN	60.15	60.06	58.94	57.71	58.86	58.78	≥ 47	≥ 51
IV (G I2.100 g^**−**1^ OIL)	43.12	44.06	56.64	70.36	57.51	58.39	-	≤ 120
SV (mg KOH g^**−**1^)	199.83	197.25	198.31	198.01	198.41	198.64	-	-
DENSITY (kg L^**−**1^)	0.87	0.87	0.87	0.88	0.87	0.87	0.85–0.9	-
CP (°C)	18.62	18.58	18.01	17.39	17.97	17.93	-	-
HHV (Mj kg^**−**1^)	39.25	39.28	39.57	39.89	39.59	39.61	-	-
LCSF (wt %)	0.14	0.22	0.172	0.14	0.13	0.15	-	-
CFPP (°C)	− 16.03	− 15.77	− 15.94	− 16.04	− 16.05	− 16.01	− 13 to − 5	≤ 5/ ≤ − 20

ASTM International (formerly American Society for Testing and Materials)-EN 14,214, The European biodiesel specification

The average degree of unsaturation of *S*. o*bliquus* (0.77) resulted in a higher iodine number but not more than 120 for high-quality biodiesel. Because the climate conditions in the USA and Europe differ, there are no clear guidelines for the cloud point (CP) (Knothe 2011). Optimum CP is the temperature that leads to wax cloud formation in the fuel. *C*. *reinhardtii* exhibited high CPs around 18.62 °C, enhancing biodiesel suitability under colder conditions. Fatty acid profile significantly affected the biodiesel quality (Ashour et al. 2019; Wang et al. 2018). The content of high saturated and low unsaturated fatty acids in our study was preferable to achieve good-quality biodiesel because saturated fatty acids have an advantageous effect on the transesterification and the quality of biodiesel (Hu et al. 2008).

In addition, a high CN value is preferable for biodiesel production, where a high value is proportionate with the ignition quality and oxidative stability and achievable at a lower unsaturation degree (Ma et al. 2013). On the contrary, a lower IV value is favorable for biodiesel production, and the lower IV value improves oxidative stability during prolonged storage (Yodsuwan et al. 2017). The SV values can reflect the highest fatty acid content.

Moreover, the high content of saturated fatty acids may decrease the CFPP properties of biodiesel because SFAs have significantly higher melting points than those of USFAs (Francisco et al. 2010). Therefore, *C*. *reinhardtii*, *S*. *obliquus*, and *M*. *braunii* demonstrated appropriate biodiesel properties in accordance with international standards, which could compete with fossil diesel.

## Conclusion

Based on the results, wastewater recycling and reuse can be accomplished through bioremediation using various algal species such as *Chlamydomonas reinhardtii*, *Scenedesmus obliquus*, and *Monoraphidium braunii*. It is also concluded that because all algal species showed higher biomass and lipid productivities, they are a feedstock for biodiesel production. Research in the future should focus on the bioengineering of microalgae for the high lipid production and accumulation of fatty acid because it would be of highly promising way to meet the demand for energy using microalgae as a feedstock for third-generation biofuel.

## Data Availability

The datasets used and/or analyzed during the current study are available from the corresponding author upon reasonable request.
